# Donor human milk programs in German, Austrian and Swiss neonatal units - findings from an international survey

**DOI:** 10.1186/s12887-020-02137-2

**Published:** 2020-05-19

**Authors:** Daniel Klotz, Stefanie Jansen, René Glanzmann, Nadja Haiden, Hans Fuchs, Corinna Gebauer

**Affiliations:** 1 Center for Pediatrics and Adolescent Medicine, Department of Neonatology, Medical Center – University of Freiburg, Faculty of Medicine, University of Freiburg, Freiburg, Germany; 2 Department of Neonatology, University Children’s Hospital, Basel, Switzerland; 3 Department of Pediatrics and Adolescent Medicine, Division of Neonatology, Pediatric Intensive Care and Neuropediatrics, Medical University of Vienna, Vienna, Austria; 4 Department of Womens’ and Children’s Health, Division of Neonatology, University Children’s Hospital, Leipzig, Germany

**Keywords:** Breast milk feeding, Donor, Human milk, Milk bank, Neonate, Preterm, Screening

## Abstract

**Background:**

Donor human milk (DHM) has been recommended for premature infants if mothers’ own milk is not available. The aim of this study was to increase the knowledge about the utilization rate and handling of DHM among neonatal units in Germany, Austria und Switzerland.

**Methods:**

Online survey of utilization rates and handling practices of DHM of all neonatal units within Germany, Austria and Switzerland providing care for premature infants less than 32 weeks of gestation.

**Results:**

DHM utilization rate of 35% is low (50/142) within those 54% of units that responded to our survey (142/261). Only 26/50 units have DHM routinely integrated into their nutritional management protocols. Lacking access and difficult procurement were cited as the main obstacles for not using DHM. However, eight out of ten respondents currently not using DHM would like to introduce DHM in their unit if available. There were differences in most aspects of DHM handling including donor recruitment and screening, testing and treatment of milk microbiota and commencement of DHM utilization. Breastmilk feeding rates were increased in units utilizing DHM compared to those not utilizing DHM.

**Conclusions:**

DHM is underutilized in most neonatal units caring for premature infants within participating countries. Lacking access to DHM represents the main barrier for utilizing DHM for premature infants.

## Background

Donor human milk (DHM) is recommended for feeding premature infants when mothers’ own milk is unavailable or not fit for consumption [1, 2]. Donor human milk banks (DHMB) are the means by which DHM may be made available for premature infants. Subsequently, the number of DHMB is increasing in many parts of the world. Several national human milk bank associations described their DHMB networks and detailed their respective mode of operations [3, 4].

However, the knowledge about the actual DHM utilization rates in neonatal intensive care units providing care for very premature infants is limited [5–7]. The aim of this study is to provide an overview about the actual utilization rate of DHM, the procurement and handling of DHM, the implementation of feeding strategies using DHM or to identify the barriers to its use within German, Swiss and Austrian neonatal units. DHM programs and DHMB within these countries are established, operated and funded exclusively by individual neonatal departments setting their own policies concerning procurement and handling of human donor milk.

These data are needed to inform health care professionals, authorities and stakeholders about the extent and practice of current DHM programs within the participating countries, to support them to establish or evaluate local or national guidelines concerning DHM utilization and DHM handling and to improve the availability of DHM for preterm infants [8–10].

## Methods

We sent a stratified online questionnaire to neonatologists within every neonatal unit that was providing care to preterm infants of less than 32 weeks of gestational age in Germany, Austria and the German speaking part of Switzerland.

The questionnaire was developed by the authors who are experienced in managing DHMB within their own neonatal departments and contained a maximum of 21 questions, depending on the strata. The questionnaire was pre-tested amongst neonatologists experienced with managing DHM programs. Participants were identified by personal knowledge or by internet research and consisted of individual neonatologists either in charge of the respective neonatal unit (i.e. the head of the neonatal department) or in charge of the DHM program of a respective neonatal unit. They were provided with information about the purpose of this study, the process of data collection and the intended publication of anonymized data. By replying to our survey, the contacted individuals consented to participate in this study. Withdrawal of their consent or supplied data and thus of participation, was possible at any time.

We asked participants to provide unit specific policies concerning the use of DHM, handling routines, the source of DHM and the overall breastfeeding rates at discharge for each unit. Screening of donors and donated milk, exclusion and reimbursement of donors were also surveyed. We inquired about the participant’s personal reasons to support the use of DHM in their unit. Rates of any or exclusive breast milk feeding (BMF) at discharge were sought. Barriers to prevent the use of DHM were enquired from those participants not utilizing DHM in their unit.

Data were collected from June 2016 to December 2018. The ethics committee of the Albert-Ludwigs-University, Freiburg, Germany, approved this study (No. 484/16).

We performed a descriptive analysis reporting quantitative data as mean and standard deviation or median and interquartile range where applicable. Categorical variables are presented in absolute numbers and percentages. The denominator represents the number of replies of any given questions to account for skipped questions. We applied a Wilcoxon rank sum test to compare unit size and breastfeeding rate between units and considered a *p*-value < 0.05 to be significant (GraphPad Prism V8, GraphPad, San Diego, CA).

## Results

We contacted neonatologists from 261 different units and 142 of those replied (54%). One hundred and three of the participating units provided the highest level of neonatal care (level III) and 39 units provided level II neonatal care (Table 1).

**Table 1:** Use of Donor human milk in participating neonatal units within Germany, Austria and Switzerland^a^

	Germany		Austria		Switzerland^a^	
Level of neonatal care^b^	Level III	Level II	Level III	Level II	Level III	Level II
Contacted centers (n)	165	58	7	17	7	7
	n (%)	n (%)	n (%)	n (%)	n (%)	n (%)
Response rate	91 (58)	27 (47)	6 (86)	6 (35)	6 (86)	6 (86)
No use of DHM	65 (72)	18 (67)	1 (17)	3 (50)	0 (0)	5 (83)
Use of DHM	26 (28)	9 (33)	5 (83)	3 (50)	6 (100)	1 (25)
Routinely^c^	13 (50)	1 (11)	4 (80)	2 (67)	6 (100)	0 (0)
Regularly	2 (8)	1 (11)	0 (0)	0 (0)	0 (0)	0 (0)
Occasionally	4 (15)	4 (44)	0 (0)	0 (0)	0 (0)	0 (0)
Rarely	7 (27)	3 (33)	1 (20)	1 (23)	0 (0)	1 (100)

^a^German speaking part of Switzerland

^b^Level III = level of maximum care

^c^as part of a standardized feeding regimen

*DHM* donor human milk

The median (IQR) number of very low birth weight infants (VLBW) per unit with a birth weight < 1500 g was 52 (36–72) in the year prior to the survey participation.

### Utilization of donor human milk

Any DHM was utilized in 50/142 neonatal units (35%). Within the year of participation, the median (range) number of neonates receiving any DHM per unit was 20 (2–59). Those units were caring for a median (IQR) of 61 (50–87) VLBW in the year prior to the survey participation, which compared to a median of 50 (33–67) VLBW in those 92 units that were not utilizing any DHM (*p* = 0.001).

DHM feeding was commenced either immediately after birth (*n* = 29), when mothers’ own milk was not available after a few days of life (*n* = 3), or commencement of feeding with DHM was decided on an individual basis (*n* = 13).

DHM was acquired from different sources. Most neonatal units with DHM programs operated an institutional DHMB (*n* = 27). In the remainder DHM was provided as a direct milk donation from another mother on the neonatal ward (*n* = 10) whereby the DHM was handled on the neonatal ward lacking the infrastructure and service of a dedicated DHMB. In some cases neonatal units without an own DHMB and that were not performing direct milk donations within their unit (*n* = 11) purchased DHM from other neonatal units, all of which operated a DHMB (*n* = 7). None of the neonatal units purchased commercially available DHM products within their year of survey participation.

No neonatal unit (and their respective DHMB) distributed DHM to private non-hospitalized individuals.

**Fig. 1:**
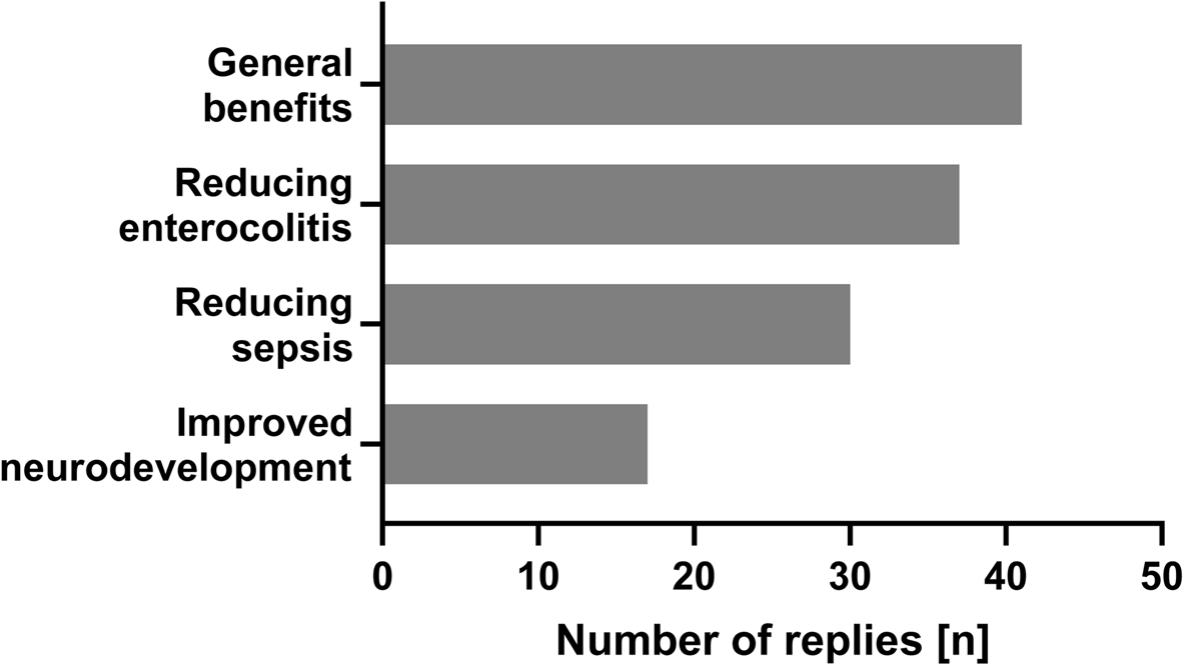
Reasons for using donor human milk amongst participants (multiple replies possible)

The main reasons to prefer DHM over preterm formula was improved neonatal short term outcome parameters (Fig. 1). Explicit parental request was cited by two participants as additional reason to feed DHM.

**Fig. 2:**
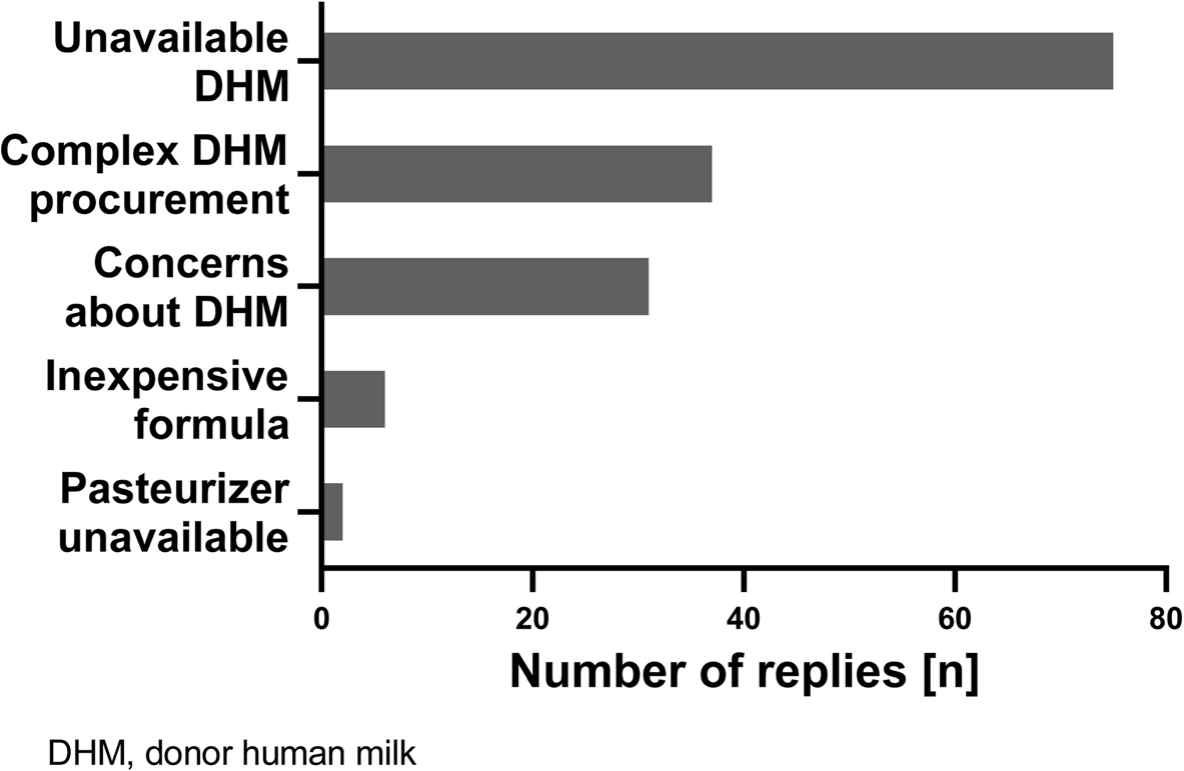
Reasons for not utilizing donor human milk amongst participants (multiple replies possible)

Non-availability of DHM and the complex process of procuring DHM were the main reasons for not utilizing DHM but general concerns about the use of DHM were also voiced (Fig. 2). However, eight out of ten respondents that did not have access to DHM would like to introduce DHM in their unit once it would become available, citing reasons similar to those participants already utilizing DHM.

### Donor recruitment and donor screening

In 24 units donors are recruited amongst lactating mothers from other infants within the neonatal unit itself or the respective children’s hospital (e.g. mothers of infants suffering from congenital heart disease), recruited amongst lactating mothers from the community not being connected to the respective hospital (external milk donation, *n* = 21) or recruited from both donor pools (*n* = 15).

A combination of a health history questionnaire regarding lifestyle, health indicators, medical and travel history, and serological testing to screen for donor eligibility was applied by all neonatal units that were procuring DHM (Table 2).

**Table 2:** Items for screening for donor eligibility

	Screening items
**Health history and lifestyle** **questionnaire (*n*** **= 37)**	n (%)
Chronic illness or long term medication	37 (100)
Nicotine abuse	37 (100)
Alcohol consumption	37 (100)
History of drug abuse	35 (95)
Promiscuity	29 (78)
Frequent consumption of caffeine	27 (70)
Special diets (vegan, vegetarian)	24 (65)
**Serological Screening (*n*** **= 40)**	
HIV 1 and 2	40 (100)
Hepatitis B	40 (100)
Syphillis	33 (83)
Hepaptitis C	32 (80)
CMV	30 (75)
HTLV 1 and 2	10 (25)

*CMV* cytomegalovirus; *HIV* human immunodeficiency virus; *HTLV* human T-lymphotrophic virus

Additionally, according to individual participants’ comments, donors were questioned for any treatment with blood products or immunizations with live vaccines, international travel to certain geographic areas, new skin tattoos, permanent make up or piercings up to 6 months prior to DHM donation but these items were not systematically surveyed by our questionnaire. Donors to some neonatal units were tested for nicotine (*n* = 3), recreational drugs (*n* = 5), medication levels (*n* = 5) or alcohol levels (*n* = 2).

Actual donor expenses related to the donation, such as travel costs, were reimbursed by 12 units. In no instances were donors paid for sharing their milk.

### Screening and handling of donor human milk

Donor milk was screened for bacterial count by 31/40  units. Screening was performed daily for every single bottle or pooled samples of DHM (*n* = 12), once a week (*n* = 10) or as random samples (*n* = 9). However, according to our survey DHM was not tested for bacterial contamination in nine cases. Post-pasteurization cultures of DHM and cytomegalovirus studies from DHM were rarely performed (*n* = 4). DHM was never tested for milk adulteration, e.g. adding water or non-human milk to DHM or for toxicological substances, e.g. alcohol or recreational drugs.

Depending on the bacterial content, DHM was left untreated (i.e. unpasteurized after being refrigerated and frozen) in 7/41 units, Holder pasteurized (i.e. DHM heated at 62.5 °C for 30 min) in 25/41 units, subjected to short-time pasteurization (i.e. 62 °C for 5 s, *n* = 2) or subjected to freeze-thawing (*n* = 11) before being distributed to preterm infants. Only one unit used DHM that has never been frozen and remained unpasteurized after cultural testing for bacterial count and bacterial identification.

### Lactation consultation and breast milk feeding

Lactations consultants were available in all but one unit. Rates of any BMF and for exclusive BMF at discharge from neonatal care were estimated by the participants for their respective unit. Rates of any BMF for preterm infants < 1500 g birthweight at discharge were increased in those units utilizing DHM (*n* = 45) compared to those units (*n* = 91) that are not utilizing DHM (median any BMF rate 71–80% versus 61–70%, *p* = 0.0008). Estimated rates for exclusive BMF at discharge were also increased in those units supplying DHM compared to those not utilizing DHM (median exclusive BMF rate 51–60% versus 41–50%, *p* = 0.019).

## Discussion

Sixty-five percent of those neonatal units that were participating in our survey did not utilize DHM in their nutritional management of very premature infants. Only half of the units that were feeding DHM used it as part of routine nutritional management, and in a third of units, DHM appeared to be used on a case by case basis only. Neither the overall utilization rate nor the implementation in those units feeding DHM reflects the actual recommendations concerning the use of DHM for premature infants [1, 2]. This is in line with previous reports from other health care systems and underlines the need to improve the utilization rate of DHM and its implementation in clinical care [6, 7, 11].

Interestingly, some of the respondents that performed direct milk donations within their own unit did not consider themselves as maintaining a DHM program. However, the need for obtaining informed consent from donors and parents of recipients, screening of donors and donated milk, preparation, distribution and tracking of DHM applies irrespective of the source of DHM. Therefore, any distribution of DHM within a health care facility may be considered as “milk banking” and should be subjected to adequate rigorous quality management according to the respective recommendations or regulations [12–14].

DHM screening for adulteration or substance abuse was not applied by our participants, this emphasizes the importance of donor screening and selection, especially when external milk donations are accepted. There was no reimbursement for milk donations to the non-profit DHM programs provided by neonatal departments in our cohort, this may reduce the financial incentive for milk adulteration that has been reported from commercially oriented milk sharing models [15].

Pasteurization of DHM is recommended to prevent the transmission of potentially harmful microbiota to the premature recipients [12, 16, 17]. The adverse impact of Holder-pasteurization on the quality of banked DHM is well known but alternatives to Holder pasteurization are limited [18, 19]. Freeze-thawing and short time pasteurization as performed in some units may not effectively inactivate cytomegalovirus or sufficiently reduce bacterial counts [20, 21]. Some units are dispensing unpasteurized DHM based on maternal cytomegalovirus (CMV) serostatus and on DHM bacterial counts for which many different threshold levels have been described and therefore remain somewhat arbitrary [22, 23].

Lactation and breastfeeding support are the prerequisites for any DHM program. All efforts must have been undertaken to provide mothers own milk first before considering an infant as a suitable DHM recipient. All but one unit offered such lactation support. However, we did not enquire about the level of expertise of these lactation consultants. Furthermore, lactation support should be available at all times but the number of lactations consultants needed to staff an effective lactation program remains to be determined [1, 24].

Estimated exclusive and any breastmilk feeding (BMF) rates at discharge did not indicate lower BMF rates in participating units utilizing DHM compared to those not utilizing DHM. We acknowledge the limited methodology, i.e. estimation, for assessing BMF rates. Our results however, are in line with previously published results that did not show decreased BMF rates in neonatal units offering DHM service [25, 26]. Nevertheless, the introduction of DHM may be detrimental to BMF efforts if mothers own milk is not adequately prioritized [27].

Lacking access to DHM was the main obstacle to utilize DHM and most participants would introduce DHM in their unit if accessible. Objections against the use of DHM were also raised by some respondents. These objections were not specified by our survey but should be addressed to understand the care providers’ concern and to identify further barriers for the use of DHM.

Some respondents purchased DHM from other neonatal departments. This may increase short-term availability and utilization of DHM but questions of inter-departmental DHM sharing (liability, regulatory framework and sustainability) remain. Costs of processing DHM are reported to exceed comparable costs for feeding mother’s own milk and preterm formula considerably [28]. Although cost effectiveness of DHM has been repeatedly demonstrated in other health care settings reimbursement for the procurement of DHM has not been established within the participating countries which may further limit the neonatal departments’ access to DHM [29].

### Limitations

DHM utilization rate and handling procedures for DHM might have changed within the data acquisition period of this survey. However, this does not change our main finding of underutilization of DHM and our data may still provide a guiding framework for establishing DHM programs if national guidelines are not available. At the time of our survey the recently published guidelines of the European Milk Bank association were not available [12]. Therefore it would be worthwhile to review the variability of DHM handling practice and utilization over time. We aimed to distribute a concise and time efficient questionnaire. Therefore, we were not able to survey all different variations of DHM handling routines or to assess the percentage of eligible infants receiving DHM within a given unit. This may need a more in-depth analysis focusing on neonatal units DHM policies by other methods. We surveyed the German speaking part of Switzerland only, therefore the degree to which these results can be generalized to the whole country is limited and due to a limited participation rate we might have underestimated the true extent of DHM utilization. Nevertheless, 27 of 33 neonatal units with officially listed DHMB within the three countries (as of December 2018) participated in this survey. Therefore, we included most of those units regularly handling and utilizing DHM.

## Conclusions

Most participants would like to utilize DHM but lack access to DHM resulting in an underutilization of DHM within most German, Austrian and Swiss neonatal units compared to the existing recommendations. These findings highlight the need to increase accessibility to DHM for premature infants [8].

## Data Availability

All data generated or analyzed during this study are included in this published article.

## Abbreviations

BMF: Breast Milk Feeding;
CMV: Cytomegalovirus
DHM: Donor Human Milk
DHMB: Donor Human Milk Bank
